# Robotic versus laparoscopic right colectomy: a meta-analysis

**DOI:** 10.1186/1477-7819-12-274

**Published:** 2014-08-28

**Authors:** Huirong Xu, Jianning Li, Yanlai Sun, Zengjun Li, Yanan Zhen, Bin Wang, Zhongfa Xu

**Affiliations:** Department of Colorectal Cancer Surgery, Shandong cancer hospital, 440 Jiyan Road, Jinan, 250117 China; Shandong Academy of Medical Sciences, Jingshi Road, Jinan, 250062 China; Department of Anesthesiology and Operation, Affiliated Hospital of Shandong Academy of Medical Sciences, 38 Wuyingshan Road, Jinan, 250031 China; Department of Gastrointestinal surgery, Affiliated Hospital of Shandong Academy of Medical Sciences, 38 Wuyingshan Road, Jinan, 250031 China

**Keywords:** Da Vinci surgical system, Laparoscopic surgery, Right colectomy, Meta-analysis

## Abstract

**Background:**

The objective of this meta-analysis was to compare the clinical safety and efficacy of robotic right colectomy (RRC) with conventional laparoscopic right colectomy (LRC).

**Methods:**

A literature search was performed for comparative studies reporting perioperative outcomes of RRC and LRC. The methodological quality of the selected studies was assessed. Depending on statistical heterogeneity, the fixed effects model or the random effects model were used for the meta-analysis. Operative time, estimated blood loss, length of hospital stay, conversion rates to open surgery, postoperative complications, and related outcomes were evaluated.

**Results:**

Seven studies, including 234 RRC cases and 415 conventional LRC cases, were analyzed. The meta-analysis showed that RRC had longer operative times (*P* < 0.00001), lower estimated blood losses (*P* = 0.0002), lower postoperative overall complications (*P* = 0.02), and significantly faster bowel function recovery (*P* < 0.00001). There were no differences in the length of hospital stay (*P* = 0.12), conversion rates to open surgery (*P* = 0.48), postoperative ileus (*P* = 0.08), anastomosis leakage (*P* = 0.28), and bleeding (*P* = 0.95).

**Conclusions:**

Compared to LRC, RRC was associated with reduced estimated blood losses, reduced postoperative complications, longer operative times, and a significantly faster recovery of bowel function. Other perioperative outcomes were equivalent.

## Background

The da Vinci robotic surgical system was a significant technological advancement in minimally invasive surgery. It has been proved to be safe and successful in colorectal surgical operations. Minimally invasive robotic surgery has many advantages, such as three-dimensional high-definition field of view, tremor filtration, augmented dexterity, capability of telesurgery, and so on. Since Weber *et al*. first reported the robotic colectomy surgery in 2002 [1], more and more surgeons were willing to use this technology. Studies have revealed the feasibility and safety of both robotic right colectomy (RRC) [2, 3] and laparoscopic right colectomy (LRC) [4, 5]. There are several studies comparing the outcomes of RRC against standard LRC, but no meta-analyses have been conducted to compare and integrate the results of these studies. The objective of this meta-analysis was to compare the safety and efficacy of RRC versus conventional LRC.

## Methods

### Information sources and search

A literature search of the Medline, EMBASE, and Ovid databases for studies that compare clinical outcomes of RRC against LRC was performed. The abstracts published at major international conferences were also manually searched. In addition, the references listed in the articles that were included were manually searched for additional studies. The last search was performed in November 2013. There were no language restrictions. ‘Robotic/robotic assisted’, ‘right colectomy’, and ‘robotic/robotic assisted vs laparoscopic right colectomy’ were employed as search terms, and both free text and Medical Subject Headings (MeSH) were used.

### Study selection and quality assessment

Full-text articles of relevant studies were obtained and independently assessed by two authors (XHR and SYL) to determine their criteria for inclusion. Disagreements on inclusion were solved through discussions and, if necessary, a third independent author (XZF) was involved. To evaluate the quality of the identified studies, the Jadad scale [6] was used for assessing randomized studies, and ‘Methodological Items for Non-Randomized Studies’ [7] was used to assess non-randomized studies.

### Criteria for inclusion and exclusion

A study had to fulfill the following criteria for inclusion: (1) Randomized and non-randomized studies that compared the perioperative outcomes of RRC and LRC, regardless of the diseases of the right-side colon; (2) if the same institution and/or authors reported more than one study, the higher-quality study or the most recent publication was included; And (3) studies that were included had to report at least one of the following outcomes: operative time, estimated blood loss, length of hospital stay, conversion rate to open surgery, postoperative complications, and related outcomes.

The reasons for exclusion were the following: (1) the perioperative outcomes and patient characteristics were not reported clearly; (2) there were overlaps between authors or institutions in the published literature; or (3) studies lacked controls.

### Statistical analysis

The meta-analysis was performed using the Review Manager software (RevMan, version 5.2, Copenhagen: Nordic Cochrane Centre, Cochrane Collaboration, 2012) that was provided by the Cochrane Collaboration. Continuous variables were pooled using the mean difference (MD) with a 95% confidence interval (95% CI), and dichotomous variables were pooled using the odds ratio (OR) with a 95% CI. If studies reported only the median, range, and size of the trial, the means and standard deviations were calculated according to Hozo *et al*. [8]. If data reported only the medians, this parameter was included. Statistical heterogeneity was evaluated by I^2^, and it was considered to be high if the I^2^ statistic was greater than 50%. The fixed effects model was used for studies with low or moderate statistical heterogeneity, and the random effects model was used for studies with high statistical heterogeneity. Sensitivity analysis was performed by repeating the meta-analysis on the studies that were excluded.

## Results

### Eligible studies

**Figure 1:**
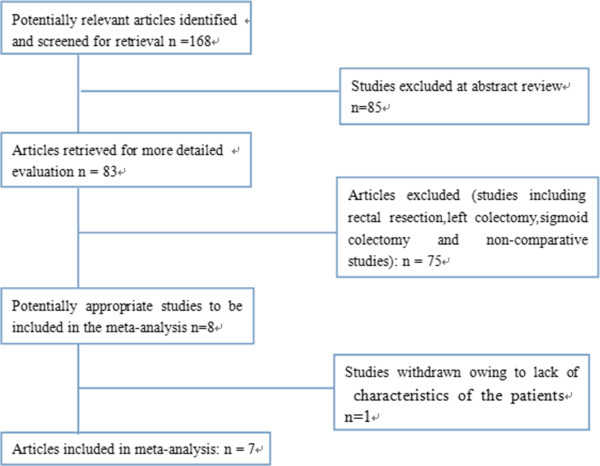
**Flow diagram of study selection for the meta-analysis.**

Following the search terms, 168 publications were initially retrieved. After carefully screening the titles, abstracts, and full text, eight comparative studies [9–16] remained in the analysis. However, one comparative study by Shin [9] was excluded because patient characteristics were not clearly reported in the right-sided colectomy subgroup and the study appeared noticeably different from the other studies. Finally, seven studies [10–16] that met all inclusion criteria were entered into this meta-analysis (Figure 1). These seven studies involved a total number of 649 patients: 234 in the RRC group and 415 in the LRC group. Of the seven studies, six were non-randomized controlled trials (NRCTs) and one was a randomized controlled trial (RCT). The baseline characteristics and quality assessment of these seven studies are listed in Table 1. The robot-assisted hybrid technique was involved in all studies. Five studies were conducted in the United States [10, 12–15], one in Korea [11], and one in Italy [16]. The quality of all the studies was satisfactory. The results showed that RRC had longer operative times, lower estimated blood losses, shorter hospital stays, lower overall postoperative complications, and a significantly faster bowel function recovery. Outcomes between RRC and LRC are listed in Table 2. There were no statistical differences in the sex composition (*P* = 0.65) and body mass index (BMI) (*P* = 0.13). However, the mean age was found to be statistically significant in favoring the RRC group (*P* = 0.001).

**Table 1:** **Characteristics of the seven studies selected for inclusion in the meta-analysis**

Study	Country	Group	Patients	Mean age	Mean BMI	Sex (M: F)	Study type	Anastomosis technique	Minors
Lujan*et al*. [10]	United States	RRC	22	71.88 ± 9.0	31.44 ± 6.02	8:14	PNR	Intracorporeal and Extracorporeal	16/24
		LRC	25	72.6 ± 11.1	27.88 ± 6.1	10:15			
Park *et al*. [11]	Korea	RRC	35	62.8 ± 10 · 5	24.4 ± 2.5	14:21	RCT	Intracorporeal and	3/5
		LRC	35	66.5 ± 11 · 4	23.8 ± 2.7	16:19		Extracorporeal	
deSouza *et al*. [12]	United States	RRC	40	71.35 ± 14.	27.33 ± 5.22	22:18	PNR	Extracorporeal	17/24
		LRC	135	65.32 ± 18	26.57 ± 6.39	62:73			
Deutsch*et al*. [13]	United States	RRC	18	65.2 ± 12	25 ± 3.8	6:12	PNR	Extracorporeal	17/24
		LRC	47	70.8 ± 14.6	28 ± 6.5	22:25			
Rawlings *et al*. [14]	United States	RRC	17	64.6 ± 11.7	25.7 ± 4.3	8:9	R	Intracorporeal	14/24
		LRC	15	63.1 ± 17.5	28.3 ± 6.4	6:9			
Casillas [15]	United States	RRC	52	65 ± 12	26.9(25.6-28.3)	25:27	PNR	Extracorporeal	14/24
		LRC	110	71 ± 12	27.0(26.128.1)	79:41			
Morpurgo [16]	Italy	RRC	48	68 ± 8	25 ± 3.5	27:21	PNR	Intracorporeal	16/24
		LRC	48	74 ± 11	28 ± 4	16:32			

RRC, robotic right colectomy; LRC, laparoscopic right colectomy; PNR, prospective not randomized; R, retrospective; RCT, randomized controlled trial.

**Table 2:** **Comparatives outcomes between RRC and LRC**

	No of studies	RRC	LRC	MD/OR	95% CI	P	I ^2^
**Characteristics**							
Age	7	234	415	-3.21	[-5.16 - –1.26]	0.001	65%
Gender ratio	7	234	415	0.93	[0.67 - 1.29 ]	0.65	62%
BMI	7	234	415	-0.54	[-1.24 - 0.16]	0.13	77%
**Intraoperative outcomes**							
Operative time (minutes)	7	234	415	51.57	[28.82 - 67.66]	<0.00001	90%
Blood loss (mL)	6	186	367	-18.79	[-28.7 - –8.88]	0.0002	20%
Conversion to open surgery	5	168	320	0.69	[0.26 - 1.89]	0.48	7%
**Postoperative outcomes**							
Hospital stay (days)	7	199	334	-0.41	[-0.93 - 0.1]	0.12	35%
Total complication	7	232	415	0.62	[0.42 - 0.92]	0.02	0%
Anastomosis leakage	7	232	415	0.55	[0.19 - 1.61]	0.28	21%
Postoperative ileus	7	232	415	0.53	[0.25 - 1.08]	0.08	0%
Bleeding	7	232	415	0.97	[0.37 - 2.57]	0.95	2%

### Operating time

**Figure 2:**
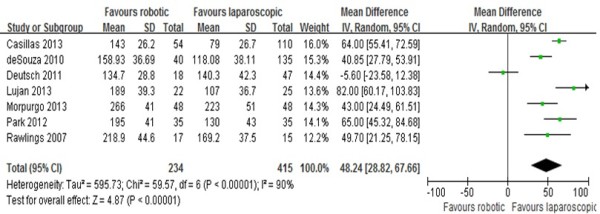
**Analysis of the operating time.** Forest plot of comparison between robotic right colectomy and conventional laparoscopic right colectomy. instrumental variables (IV) is used to estimate causal relationships when controlled experiments are not feasible.

The operating time was reported in all seven studies [10–16], six of which supported the finding that operating times were longer in RRC. The study by Deutsch *et al*. [13] was the only one showing no differences between the two approaches. In this study, undocking time was not included in the operating time. Because of the high heterogeneity (I^2^ = 90%) of these studies, the random effects model was used for the meta-analysis. The results show that the RRC group had a significantly longer operating time compared to the LRC group (MD = 48.24; 95% CI: 28.82 - 67.66; *P* < 0.00001). Analysis excluding the data from the study by Deutsch *et al*. [13] still shows a significant statistical difference in operating time that favors RRC (MD = 57.83; 95% CI:51.88 - 63.78; *P* < 0.00001), and the heterogeneity is still high (I^2^ = 90%;Figure 2).

### Length of hospital stay

**Figure 3:**
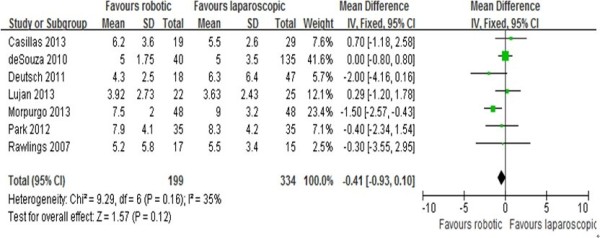
**Analysis of the length of hospital stay.** Forest plot of comparison between robotic right colectomy and conventional laparoscopic right colectomy. instrumental variables (IV) is used to estimate causal relationships when controlled experiments are not feasible.

The length of hospital stay was reported in all seven studies [10–16]. Results of the meta-analysis show no differences between the RRC and the LRC groups (MD = -0.41; 95% CI: -0.93 -0.1; *P* = 0.12), with a low heterogeneity (I^2^ = 35%;Figure 3).

### Conversion to open surgery

The conversion rate to open surgery was reported in five studies [10–12, 14, 15]. Results of the meta-analysis show no differences between the RRC and the LRC groups (OR = 0.69, 95% CI: 0.26 - 1.89; *P* =0.48), with a low heterogeneity (I^2^ = 7%).

### Estimated blood loss

**Figure 4:**
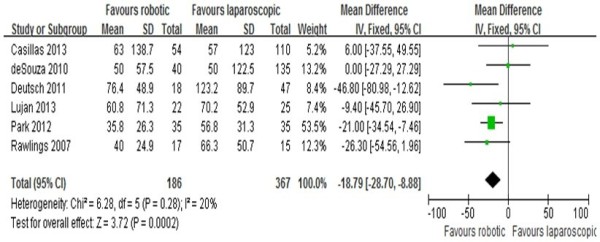
**Analysis of the estimated blood loss.** Forest plot of comparison between robotic right colectomy and conventional laparoscopic right colectomy instrumental variables (IV) is used to estimate causal relationships when controlled experiments are not feasible.

The estimated intraoperative blood loss was reported in six studies [10–15]. Results of the meta-analysis show that intraoperative estimated blood loss was significantly lower in patients from the RRC group as compared to patients undergoing LRC (MD = -18.79; 95% CI: -28.7 - –8.88; P = 0.0002), with a low observed heterogeneity (I^2^ = 20%; Figure 4).

### Time to bowel function recovery

Time to bowel function recovery was reported in three studies [10, 11, 13]. Results of the meta-analysis show that bowel function recovered much faster in patients undergoing RRC than in those undergoing LRC (MD = -0.79; 95% CI: -1.10 - –0.48;, *P* < 0.000001), with a low heterogeneity (I^2^ = 26%).

### Overall postoperative complications

**Figure 5:**
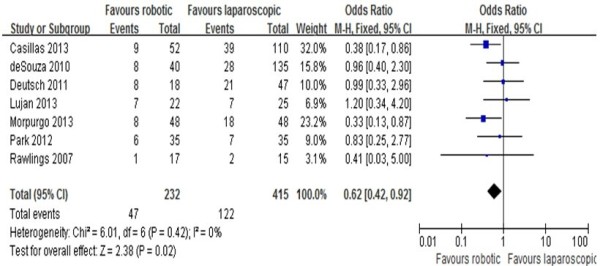
**Analysis of overall postoperative complications.** Forest plot of comparison between robotic right colectomy and conventional laparoscopic right colectomy. Meta-analyses of dichotomous variables were performed using the Mantel–Haenszel (M-H) method.

Overall postoperative complications were reported in six studies [10–16]. Results of the meta-analysis show that overall complications in the RRC group were significantly fewer than in the LRC group. (MD = 0.62; 95% CI: 0.42 - 0.92; *P* = 0.02), with no observed heterogeneity (I^2^ = 0%). In terms of postoperative ileus, the meta-analysis shows no difference between the two groups (MD = 0.53; 95% CI:0.25 -1.08; *P* = 0.08) with no heterogeneity (I^2^ = 0%). The meta-analysis also shows no difference between the two groups in terms of anastomosis leakage (MD = 0.55; 95% CI: 0.19 -1.61; *P* = 0.28) and postoperative bleeding (MD = 0.97; 95% CI: 0.37 -2.57; *P* = 0.95), with a low heterogeneity (I^2^ = 21% and 2%, respectively; Figure 5).

## Discussion

After being first described in 1991, laparoscopic colorectal surgery has rapidly evolved in recent years [17]. Currently, most colorectal procedures are performed laparoscopically. Several studies show that laparoscopic colorectal surgery is associated with better short-term outcomes than open surgery, and the benefits include smaller incisions, reduced postoperative pain and duration of ileus, faster postoperative recovery, shorter hospital stay, and earlier recovery of normal activity [18, 19]. However, the laparoscopic approach has several limitations such as tremor, loss of three-dimensional view, inability to perform high-precision suturing, poor ergonomics, fixed tips, and limited movement dexterity [20]. The da Vinci surgical system has been developed to overcome such difficulties and has become increasingly popular in colorectal surgery. The system is equipped with a three-dimensional high-definition camera, and is capable of physiological tremor filtration. Furthermore, it enables three extra degrees of movement by using articulated instruments. Therefore, it can minimize the risk of injury to vessels and nerve structures and provide oncological resection capabilities [21]. Furthermore, the surgical system can also decrease the learning curve for laparoscopic surgery [22]. However, there are also limitations of this system such as the loss of haptic feedback, limited range of movement of the robotic arms and, in addition, it is time-consuming and high-cost. The limited intracorporeal range of motion is the major drawback in colectomy when operating in a large operative field [23]. Up to now, several studies [9–16] have compared the safety and efficacy of RRC and conventional LRC, but no meta-analyses were performed to compare and integrate the results of these studies. To the best of our knowledge, this is the first comprehensive meta-analysis comparing RRC and LRC.

Although RCTs are usually applied and supposed to be used [24] in meta-analyses, this is not always possible or feasible in surgical research studies [25]. Therefore, meta-analyses using NRCTs might be a good method for surgical trials, and most of the available evidence in surgery comes from non-randomized studies [7]. However, selection bias exists in these studies because most of the studies are not randomized and preoperative characteristics are not equal across some of the studies. High heterogeneity may not provide useful meta-analysis results. However, it is the impact of factors other than the surgical approach that affect these outcomes. The present meta-analysis points towards the apparent feasibility of RRC. The pooled results of the seven studies showed that RRC had longer operative times, lower estimated blood loss, shorter hospital stays, lower overall postoperative complications, and a significantly faster bowel function recovery.

A long operative time was widely reported in robotic colorectal surgery. Many factors influence operative times, and these include set-up time, docking time, learning curve, and the type of anastomosis [26]. In terms of right colectomy, either a hybrid technique or repositioning of the robotic cart is required [27]. Multiple dockings of the robotic cart and the creation of a proper surgical field are also time-consuming. The set-up time was excluded from all the seven studies included in the meta-analysis. Operative time was reported to be longer in RRC than in LRC in most of the studies, except for the study by Deutsch *et al*. [13]. This meta-analysis indicates that the operative time in patients from the RRC group was significant longer than in the LRC group. The heterogeneity of the operative time between the approaches was very high (I^2^ = 90%). The reasons for this high heterogeneity of the operating time are threefold. Firstly, different diseases of the right-side colon, such as cancer, diverticulitis, polyps, Crohn’s disease, and so on, were included in different studies. Furthermore, some studies included both benign and malignant diseases [10, 12–15], and some included only right-side colon cancer. Secondly, anastomosis techniques were different in the different studies, as shown in Table 1. Thirdly, the learning curve in RRC occurred earlier than that in the LRC procedures. Therefore, the operating surgeon was commonly relatively inexperienced in RRC procedures. However, clinical and oncological outcomes improved significantly in laparoscopic surgery with the increase of experience [28]. Furthermore, operative times in robotic colorectal surgery became shorter as the surgeon’s experience improved [29]. We believe that outcomes in robotic surgery will significantly improve in the future.

We found less estimated intraoperative blood loss in patients undergoing RRC than that in those undergoing LRC. The three-dimensional high-definition field of view and augmented dexterity enabled surgeons to detect small structures and blood vessels, which contributed to the decreased blood loss. Reduced blood is also associated with reduced hospital stay and decreased conversion rates to open surgery.

Patients undergoing RRC recover their bowel function much faster than those undergoing LRC. This may be explained by the performance of intracorporeal anastomosis in the studies that we included. Intracorporeal anastomosis is proven to have several advantages, such as lower colon mobilization, fewer complications related to the exteriorization of the mesentery, and smaller incisions to extract the specimen [30]. These advantages can minimize the damage to the intestine and enable faster bowel function recovery. Meanwhile, a small incision can reduce postoperative pain, surgical site infections, and incision hernia [31]. Additionally, the major advantages of robotics are the three-dimensional view and the endo-wrist movements that facilitate intracorporeal suturing. The robotic-assisted intracorporeal anastomosis has been compared with extracorporeal anastomosis in the study by Morpurgo *et al*. [16]. Anastomotic complications were observed in the LRC with extracorporeal anastomosis and none in the RRC. Meanwhile, the extracorporeal anastomosis group have a significantly higher risk of incisional hernia than the intracorporeal anastomosis group.

In our meta-analysis, there were significantly fewer overall complications in the RRC group, which can be explained by the higher age of patients from the LRC group. At the same time, a good field of vision and precise movements may minimize the risk of tissue injuries and, finally, lead to fewer complications. However, in terms of postoperative ileus, anastomotic leakage, and postoperative bleeding, the meta-analysis shows no differences between the two groups. More advanced studies are still needed to verify this conclusion.

A major drawback of robotic rectal surgery is its high cost. The cost was not taken into account when performing the comparison because only two studies [11, 12] provided these data. In the two studies, total costs were significantly higher for RRC than for LRC. Baek*et al*. reported that the total cost in robotic surgery was approximately 1.5 times higher than in the laparoscopic group [32]. A recent systematic review also showed that robot-assisted laparoscopic resection had significant higher costs and longer operative times than traditional laparoscopic resections, but no measurable benefits were obtained [33].

Several limitations existed in the present meta-analysis. Firstly, the studies included in this meta-analysis consisted of one randomized controlled trial and six non-randomized controlled trials, and NRCTs can bias the interpretation of results in spite of quality scores [34]. Secondly, the included studies had relatively limited numbers of patients, and it was difficult to perform subgroup analyses. Thirdly, this meta-analysis could not interpret the problems caused by confounding factors that were inherent to the included studies. Finally, it was impossible to match the characteristics of the patients in most of the studies, and heterogeneity exists in the two groups.

## Conclusions

In conclusion, this meta-analysis suggests that RRC has longer operative times, lower estimated blood losses, shorter hospital stays, lower rates of overall postoperative complications, and a significantly faster bowel function recovery. Other clinical and oncological outcomes appear to be equivalent. Future well-designed prospective RCTs are required to better define this technique.

## Abbreviations

BMI: Body mass index
LRC: Laparoscopic right colectomy
MD: Mean difference
MeSH: Medical subject headings
NRCTs: Non-randomized controlled trials
OR: Odds ratio
RCT: Randomized controlled trial
RRC: Robotic right colectomy.
